# Distinct positive and negative dimensions of psychotherapy treatment expectations across three independent samples

**DOI:** 10.1038/s41598-026-60951-7

**Published:** 2026-07-03

**Authors:** Marcel Wilhelm, Lukas Andreas Basedow, Stefan Salzmann, Anna Seewald, Ulrike Bingel, Winfried Rief

**Affiliations:** 1 Division of Clinical Psychology, Marburg University, Gutenbergstrasse 18, 35037 Marburg, Germany; 2 Faculty of Human Sciences, Division of Clinical Psychology and Psychotherapy, Vinzenz Pallotti University, Vallendar, Germany; 3 Department of Neurology, Center for Translational Neuro- and Behavioral Sciences, University Medicine Essen, Essen, Germany; 4 Translational Pain Research Unit, University Medicine Essen, Essen, Germany

**Keywords:** Treatment expectations, Psychotherapy outcomes, Mental disorders, Side effects, Depressive symptoms, Health care, Psychology, Psychology

## Abstract

Treatment expectations are central to psychotherapy outcomes but are typically assessed as a unidimensional construct focusing on anticipated improvement. This study investigated whether expectations of improvement, worsening, and side effects, as measured by the Generic Rating Scale for Previous Treatment Experiences, Treatment Expectations, and Treatment Effects (GEEE), represent distinct dimensions in psychotherapy. We examined three samples differing in treatment context and symptom burden: patients currently in psychotherapy (*N* = 102), prescreened patients awaiting psychotherapy (*N* = 83), and a community sample of former psychotherapy patients who reported current mental health problems (*N* = 219). Across all groups, expectations of improvement were distinct from expectations of worsening and side effects, whereas the latter two showed moderate overlap. Improvement expectations correlated strongly with the Credibility/Expectancy Questionnaire (CEQ), while worsening and side effect expectations did not, indicating discriminant validity. In the psychotherapy sample, depressive symptoms were negatively associated with improvement expectations and positively with worsening and side effect expectations. The findings support the GEEE as a brief and multidimensional tool that extends beyond existing measures by capturing both positive and negative facets of treatment expectations. Differentiating these domains may help identify patients at risk of disengagement and inform tailored interventions to foster more adaptive expectation profiles in psychotherapy.

**Supplementary Information:**

The online version contains supplementary material available at 10.1038/s41598-026-60951-7.

## Introduction

### Treatment expectations as a common factor in psychotherapy

Patients’ expectations about psychotherapy outcomes are beliefs regarding the consequences of treatment and have long been regarded as a core common factor. Frank^1^ emphasized the restoration of hope as essential for change, Kirsch^2^ conceptualized expectations as self-fulfilling prophecies central to placebo effects and psychotherapy. Within the common factors framework, expectations - together with alliance, empathy, and therapist genuineness - are considered universal mechanisms that contribute to positive therapy outcomes^3^.

### Positive and negative expectations and their clinical relevance

Meta-analyses of psychotherapy trials show that positive treatment expectations predict more favorable results, with small but reliable effect sizes across modalities and diagnoses^4,5^. Expectations are also intertwined with the therapeutic alliance, a robust predictor of treatment outcome itself^6^. Alliance may partially mediate the link between expectancy and outcome, suggesting that positive expectations enhance engagement and collaboration, thereby facilitating improvement^7^.

Treatment expectations are not necessarily unidimensional. Patients often hold both the expectation of improvement and concerns about worsening or side effects, and negative expectations can undermine engagement or contribute to nocebo effects. Some evidence indicates that improvement expectations are more predictive of outcomes than those of worsening^8^. Longitudinal research further suggests that it is particularly the absence of positive expectations, rather than the presence of negative ones, that predicts depressive symptoms and suicidality over time^9^. These findings emphasize that fostering positive treatment expectations may be more clinically relevant than merely reducing negative expectations. In addition, studies from various medical fields have shown that negative expectations are predictive for side-effect occurrence and can also contribute to reduced adherence and premature treatment discontinuation^10–12^. These findings lend further support to the notion that positive and negative treatment expectations may differentially contribute to therapeutic processes and outcomes.

Importantly, expectations are also malleable. They tend to increase over the course of effective therapy and vary across therapists, underscoring their dynamic and relational nature^13^. Experimental research demonstrates that therapist warmth and competence can shift negative expectations toward more positive ones^14^. Moreover, structured interventions such as optimized informed-consent procedures can foster realistic optimism, strengthen motivation, and reduce decisional conflict without inflating fears of side effects^15^.

## Measurement of treatment expectations

The most widely used instrument to assess treatment expectations is the Credibility and Expectancy Questionnaire (CEQ^16^). The CEQ captures how credible patients perceive a treatment to be and how much improvement they expect from it. However, its focus lies mainly on positive expectations, and it does not account for possible concerns about worsening or side effects. More recently, the Treatment Expectation Questionnaire (TEX-Q) was developed to provide a generic, multidimensional measure that integrates theoretical models of expectations and is applicable across medical and psychological treatments^17,18^. The TEX-Q differentiates probabilistic and value-based expectations, includes both positive and negative outcome expectations, and extends the assessment to process and control expectations. While the CEQ remains the most frequently applied tool in psychotherapy research, the TEX-Q addresses a more theory-based concept by covering a broader spectrum of expectations, though its length makes it less practical for routine use.

## The GEEE: a multidimensional approach to treatment expectations

The Generic Rating Scale for Previous Treatment Experiences, Treatment Expectations, and Treatment Effects (GEEE)^19^ was developed to complement these instruments by combining a generic, treatment-agnostic format with a structure that explicitly parallels expectations, prior experiences, and current treatment effects. The GEEE assesses the same three domains (improvement, worsening, side effects) using single-item ratings with identical wording across three modules: prior treatment experiences, current treatment expectations, and current treatment effects. This parallel structure allows direct within-person comparison between, for example, prior experiences of improvement and current improvement expectations. Prior treatment experiences in this context refer to past, completed treatments rather than ongoing therapy; the instrument can therefore be completed regardless of whether such experience is present, in which case the prior-experience items are not applicable. Large-scale analyses have shown that prior experiences of symptom improvement foster higher expectations of improvement in future treatments, while negative prior experiences primarily increase side-effect expectations^20^, and the GEEE is the first instrument to assess both sides of this relationship within a single parallel framework. While its compact format entails a trade-off in conceptual depth relative to longer multidimensional scales, the GEEE is meant to be particularly suitable for routine clinical use and for studies with constrained assessment windows. Conceptually, the GEEE is best understood primarily as a measurement architecture rather than as a new construct: a brief, parallel item structure that enables joint, within-person assessment of related expectation domains across prior experiences, expectations, and current effects, in a format suited to routine and longitudinal use. A more modest, dimension-level contribution lies in its separation of expectations of worsening from expectations of side effects, which differentiates negative expectations more finely than existing brief measures.

In psychotherapy, the concept of *side effects* refers to unintended negative consequences that occur as a result of treatment processes rather than despite them. These may include the emergence of new symptoms, interpersonal or emotional strain, or dependency on the therapist^21,22^. While side effects are well established in pharmacological contexts, they remain underrecognized in psychotherapy research and clinical practice. Importantly, expectations of such adverse effects, e.g., fearing increased distress during therapy or emotional destabilization, may influence engagement, adherence, and perceived safety of treatment. Thus, assessing side-effect expectations complements the traditional focus on anticipated improvement and offers a more comprehensive understanding of patients’ treatment outlook.

Since the GEEE’s introduction, it has been applied in a variety of clinical contexts, including Crohn’s disease^23^, endometriosis^24^, post-COVID rehabilitation^25^, open-label placebo interventions^26^, and chronic back pain trials^27,28^. These studies consistently show that positive treatment expectations predict better outcomes, whereas negative expectations, particularly side effect expectations, contribute to greater functional impairment and symptom persistence. Moreover, extensions of the GEEE framework have been used in experimental and psychometric work to explore implicit outcome expectations^29^ and the role of contextual factors such as treatment uncertainty^30^. However, despite these diverse applications, research using the GEEE in the context of mental disorders or psychotherapy has been lacking to date. The present study aims to close this gap by examining the GEEE’s structure and validity in three samples representing different stages of psychotherapy involvement. Depressive symptoms are closely linked to biases in expectations. Building on the cognitive model^31^, an expectation-focused account proposes that depression persists because negative expectations are insufficiently updated when positive events occur, due to reduced learning from prediction errors and blunted reward processing, and because patients employ cognitive immunization strategies that nullify expectation-violating experiences^32–35^. Experimental work shows that when cognitive immunization is inhibited, positive feedback can shift expectations in depressed patients, underscoring modifiability of persistent expectations^36,37^. Related evidence indicates reduced revision of negative interpretations after disconfirmatory information^38^ and deficits in processing expectation violations^39^. This framework motivates examining how depressive symptoms relate to the three GEEE dimensions, with the prediction that higher symptom severity will be associated with lower expectations of improvement and stronger concerns about worsening and side effects.

Taken together, these considerations motivate testing the distinctiveness and validity of improvement expectations, expectations of worsening, and side effect expectations in clinical and subclinical samples differing in treatment context and symptom burden. Comparing individuals currently in psychotherapy, prescreened patients awaiting psychotherapy, and former patients with self-reported mental health problems allows us to examine whether the three expectation dimensions and their associations with the CEQ generalize across samples, and whether there are correlations of expectations with depressive symptoms in the psychotherapy sample. This directly sets up our research questions regarding dimensional distinctiveness, cross-sample consistency, convergent–discriminant relations with the CEQ, and associations with depressive symptoms.

Research questions

1. Are the three dimensions of the GEEE (expectations of improvement/worsening/side effects) distinct?
2. Is this pattern consistent in three samples (individuals currently undergoing psychotherapy, potential patients on the waitlist for psychotherapy at an outpatient clinic, and former patients with self-reported mental health problems)?
3. Do correlations with the CEQ support the convergent and discriminant validity of the GEEE dimensions, i.e., do improvement expectations relate to positive treatment expectations (CEQ) while expectations of worsening and side effects show distinct, weaker, or negative associations?
4. Are depressive symptoms associated with any of the three dimensions of the GEEE?

## Methods

### Sample collection and recruitment

This study aimed to evaluate the applicability of the GEEE in mental disorders, and focused on three distinct participant groups: individuals currently undergoing psychotherapy, prescreened potential patients on the waitlist for psychotherapy at the outpatient clinic of Philipps-University Marburg, and an online sample of former psychotherapy patients who reported current mental health problems, all over the age of 18. Ethical approval was granted by the Ethics Committee of Philipps University of Marburg (Reference numbers: 2021-76k, 2022-11k, 2022-02k). The studies adhered to ethical standards, ensuring informed consent, confidentiality, and participants’ right to withdraw.

### Data collection

Data collection was conducted online via the SoSci Survey platform^40^. This facilitated the secure collection of participant data and test materials. Sociodemographic information, mental health status, and treatment history were collected from participants across all groups. The psychotherapy and waitlist samples completed the questionnaire directly at the outpatient clinic on a tablet, while the former patient sample participated online.

The waitlist sample was drawn from a separate study investigating the optimization of expectations prior to the start of psychotherapy (manuscript under review). Participants in this sample were patients on a waitlist for psychotherapy at the outpatient clinic of Marburg University, meaning that the upcoming psychotherapy for which their expectations were assessed had not yet started. This does not preclude prior psychotherapy experience. For the present analyses, only baseline data of the parent study were used, ensuring that no experimental manipulation from that study had taken place at the time of GEEE assessment. The former patient sample was originally recruited as part of a larger online study on outcome expectations in psychotherapy^29^. In the current work, we reanalyzed a subset of this dataset, including only former psychotherapy patients who also reported current mental health problems. This restriction was chosen for two reasons: first, expectations regarding psychotherapy are only meaningful in individuals with current mental health difficulties; second, focusing on participants with prior psychotherapy experience increases comparability with the other two samples and raises the likelihood that self-reported problems reflect clinically relevant symptomatology.

### Instruments

Generic Rating Scale for Previous Treatment Experiences, Treatment Expectations, and Treatment Effects (GEEE): The GEEE is a concise yet comprehensive instrument^41^. Participants rate their expectations of improvement, worsening, and side effects on a Numeric Rating Scale that ranges from 0 (indicating no improvement/worsening/side effects) to 10 (reflecting the greatest imaginable improvement/worsening/side effects). This scale allows for a nuanced understanding of individuals’ expectations regarding any given treatment, and the primary outcome variables and treatment modalities can be adapted according to the needs of the study, thus allowing comparison of treatment expectations even of different treatments (e.g.^28^). Its construction is based on the premise that these dimensions are distinct, enabling a nuanced exploration of treatment expectations and their influence on therapy outcomes. Besides expectations, the GEEE also includes the same 3 items (improvement/worsening/side effects) regarding prior experiences of the treatment and regarding current effects of the treatment. The GEEE’s economic design, comprising just three items for expectations, four items for prior treatment experiences, and three items for current treatment effects allows for its flexible application across various contexts and treatments.

Credibility/Expectancy Questionnaire (CEQ): Adapted from Devilly and Borkovec^16^, the CEQ is used to measure treatment-related credibility and expectancy, comprising six items. Four of these items are rated on a 9-point Likert scale, with responses ranging from 1 (not at all) to 9 (very much). Additionally, there are two items rated on a percentage scale from 0% to 100%, with responses recorded in increments of 10% (i.e., 0%, 10%, 20%, ..., 100%). The inclusion of the CEQ in this study aids in comparing standardized measures of treatment expectations across different participant groups, thereby providing a baseline for evaluating the construct validity of the GEEE.

Patient Health Questionnaire-9 (PHQ-9): The PHQ-9 is a widely used self-report measure of depressive symptom severity^42^. It comprises nine items that correspond to DSM-5 criteria for major depressive disorder. Each item is rated on a 4-point scale ranging from 0 (not at all) to 3 (nearly every day), referring to the frequency of symptoms during the past two weeks. Item scores are summed to yield a total score from 0 to 27, with higher scores indicating greater depressive symptom severity (commonly interpreted as minimal = 0–4, mild = 5–9, moderate = 10–14, moderately severe = 15–19, severe = 20–27). In the present study, the total PHQ-9 sum score was used to index depression severity for correlation analyses with treatment expectations and experiences.

### Statistical analyses

All analyses were conducted using R (Version 4.4.1^43^), using the *tidyverse*^44^ and *psych* packages^45^. Descriptive statistics were computed separately for each of the three samples: patients currently in psychotherapy, prescreened patients awaiting psychotherapy, and former psychotherapy patients with current self-reported mental health problems. Throughout this manuscript, the term ‘dimension’ is used in its conceptual sense, to refer to theoretically distinct content aspects of treatment expectations (i.e., improvement, worsening, and side effects), rather than in the strict psychometric sense of latent factors derived from multiple indicators. Pearson correlation coefficients were calculated to examine associations among GEEE dimensions, prior treatment experiences, and current treatment effects. For the psychotherapy sample, additional correlations with PHQ-9 scores were computed to assess associations between depressive symptoms and expectations.

Convergent and discriminant validity were evaluated by correlating GEEE improvement, worsening, and side effect expectations with the credibility and expectancy subscales of the CEQ. To assess the distinctiveness of dimensions, correlations were compared across domains, with particular attention to whether positive expectations (improvement) were independent of negative expectations (worsening, side effects).

All correlations were calculated separately for each sample to allow comparison across groups. Effect sizes were interpreted according to Cohen’s^46^ guidelines (*r* = 0.10 small, *r* = 0.30 medium, *r* = 0.50 large). For each correlation, 95% confidence intervals and exact *p* values were reported. Significance thresholds were set at *p* <0.05 (two-tailed). To evaluate whether the correlational structure of the GEEE dimensions was comparable across the three samples, we examined differences in correlation coefficients between groups. Following Cohen’s^46^ conventions for interpreting effect sizes, correlations were considered comparable when differences between groups did not exceed Δr = 0.20.

Between-group differences in expectations, prior experiences, and CEQ scores were analyzed using one-way analyses of variance (ANOVAs) with sample (psychotherapy patients, waitlist patients, former psychotherapy patients) as the independent variable. Significant omnibus effects were followed up with Tukey-adjusted post hoc comparisons. Effect sizes were expressed as eta squared (η²) and interpreted according to Cohen’s^46^ conventions (*η²* ≈ 0.01 small, 0.06 medium, 0.14 large). Homogeneity of variance was checked using Levene’s test.

## Results

### Sample characteristics

This study examined *N* = 404 participants across three samples: patients currently in psychotherapy (*n* = 102), individuals on a waitlist to receive psychotherapy (*n* = 83), and an online cohort contemplating psychotherapy (*n* = 219). The combined demographic profile revealed a predominance of women (69.9%) and an average age of 35.2 years. Notably, optimistic expectations for improvement were observed across all samples, with a mean score suggesting high anticipation of positive therapy outcomes (see Table 1).

**Table 1:** Sample characteristics.

	Psychotherapy patients (*n* = 102)	Wait list patients (*n* = 83)	Online sample (*n* = 219)	Overall (*N* = 404)
Gender				
Female	70 (68.6%)	46 (45.5%)	179 (81.7%)	295 (73.0%)
Male	29 (28.4%)	35 (35.6%)	38 (17.4%)	102 (25.2%)
Diverse	3 (2.9%)	2 (2.0%)	2 (0.9%)	7 (1.7%)
Age in years				
Mean (*SD*)	35.9 (14.4)	34.2 (13.6)	35.4 (13.3)	35.3 (13.6)
Median [Min, Max]	30.0 [19.0, 64.0]	30.0 [18.0, 73.0]	29.0 [20.0, 78.0]	29.0 [18.0, 78.0]
Not reported	1 (1.0%)	0 (0%)	0 (0%)	1 (< 0.1%)
Expectation of improvement				
Mean (*SD*)	8.40 (1.66)	8.11 (1.61)	8.10 (1.85)	8.18 (1.76)
Median [Min, Max]	9.00 [2.00, 11.0]	8.00 [4.00, 11.0]	9.00 [1.00, 11.0]	9.00 [1.00, 11.0]
Expectation of worsening				
Mean (*SD*)	2.07 (1.56)	2.57 (2.17)	2.32 (1.82)	2.30 (1.84)
Median [Min, Max]	1.00 [1.00, 8.00]	2.00 [1.00, 9.00]	2.00 [1.00, 11.0]	2.00 [1.00, 11.0]
Expectation of side effects				
Mean (*SD*)	3.47 (2.30)	3.69 (2.63)	4.27 (2.52)	3.95 (2.51)
Median [Min, Max]	3.00 [1.00, 10.0]	3.00 [1.00, 11.0]	4.00 [1.00, 11.0]	3.00 [1.00, 11.0]

### Descriptive statistics and group differences in treatment expectations

Descriptive statistics for the three samples psychotherapy patients (*n* = 102), patients on a psychotherapy waitlist (*n* = 83), and former psychotherapy patients with current mental health problems (*n* = 219) are summarized in Table 1. Across all groups, expectations of improvement were rated toward the upper end of the 0–10 scale (*M* = 8.18, *SD* = 1.76), whereas expectations of worsening (*M* = 2.30, *SD* = 1.84) and side effects (*M* = 3.95, *SD* = 2.51) were positioned in the lower-to-middle range.

One-way ANOVAs examined group differences in the three expectation dimensions and in the CEQ scales. Expectations of improvement did not differ significantly between groups, *F*(2, 401) = 1.105, *p* = 0.332, nor did expectations of worsening, *F*(2, 401) = 1.683, *p* = 0.187.

A small but significant effect emerged for expectations of side effects, *F*(2, 401) = 4.204, *p* = 0.016, *η²* = 0.020. Post-hoc Tukey tests indicated that former psychotherapy patients anticipated more side effects (*M* = 4.27, *SD* = 2.52) than psychotherapy patients currently in treatment (*M* = 3.47, *SD* = 2.30), *p* = 0.020, *d* = 0.33, reflecting a small-to-medium difference. The waitlist group did not differ significantly from either of the other two groups.

For the CEQ, a significant group effect was found for treatment credibility, *F*(2, 401) = 8.575, *p* <0.001, *η²* = 0.040. Psychotherapy and waitlist patients rated psychotherapy as more credible than former patients (both *ps* < 0.010; psychotherapy vs. former patients: *d* = 0.51; waitlist vs. former patients: *d* = 0.48). No significant group differences were observed for the CEQ expectancy scale, *F*(2, 401) = 0.229, *p* = 0.795.

To examine whether the observed group differences were attributable to the unequal gender distribution across samples, all comparisons were re-run as ANCOVAs with gender and age as covariates. The pattern of results was fully unchanged: covariate-adjusted means closely mirrored raw means across all outcomes, and significance status was identical for all five variables examined, supporting the robustness of the findings (see Supplement for details).

### Item-wise associations within GEEE

Across most samples, expectations of improvement, worsening, and side effects showed low intercorrelations, suggesting that they largely represent distinct constructs. An exception was the former patient sample, in which a small but significant negative association between expectations of improvement and worsening was observed (*r* = – 0.20, 95% CI [–0.32, − 0.07], *p* = 0.003, *n* = 219), indicating some degree of overlap in this group.

*Expectation of improvement* were positively correlated with prior experiences of improvement (in psychotherapy patients *r* = 0.26, 95% CI [0.00, 0.48], *p* = 0.048, *n* = 60, patients on a psychotherapy waitlist *r* = 0.36, 95% CI [0.10, 0.58], *p* = 0.009, *n* = 50, and former patients with self-reported mental health issues *r* = 0.50, 95% CI [0.40, 0.60], *p* <0.001, *n* = 219). Expectations of improvement were also positively correlated with current improvement effects in psychotherapy patients (*r* = 0.22, 95% CI [0.03, 0.40], *p* = 0.025, *n* = 102). In addition, expectations of improvement were slightly negatively correlated with prior experiences of worsening in former patients (*r* = – 0.15, 95% CI [–0.28, − 0.02], *p* = 0.026, *n* = 219).

*Expectations of worsening* were positively correlated with prior experiences of worsening (in psychotherapy patients *r* = 0.37, 95% CI [0.12, 0.57], *p* = 0.004, *n* = 60, patients on a psychotherapy waitlist *r* = 0.59, 95% CI [0.38, 0.75], *p* <0.001, *n* = 50, and former patients with self-reported mental health issues *r* = 0.60, 95% CI [0.51, 0.68], *p* <0.001, *n* = 219). In addition, they were positively associated with current worsening effects in psychotherapy patients (*r* = 0.47, 95% CI [0.30, 0.61], *p* <0.001, *n* = 102).

*Expectations of side effects* were positively correlated with prior side effect experiences (in psychotherapy patients *r* = 0.29, 95% CI [0.04, 0.51], *p* = 0.024, *n* = 60, patients on a psychotherapy waitlist *r* = 0.42, 95% CI [0.16, 0.63], *p* = 0.002, *n* = 50, and former patients with self-reported mental health issues *r* = 0.70, 95% CI [0.63, 0.76], *p* <0.001, *n* = 219). They were also positively associated with current side effect experiences in psychotherapy patients (*r* = 0.46, 95% CI [0.29, 0.60], *p* <0.001, *n* = 102).

*Analyses of interrelationships among dimensions* showed that prior experiences of improvement were negatively correlated with both prior worsening (*r* = – 0.42, 95% CI [–0.60, − 0.19], *p* <0.001, *n* = 60) and prior side effects (*r* = – 0.32, 95% CI [–0.53, − 0.07], *p* = 0.013, *n* = 60) in psychotherapy patients, supporting a conceptual distinction between positive and negative outcome perceptions. In patients on a psychotherapy waitlist, strong associations were observed between negative experiences, with prior worsening being strongly associated with prior side effects (*r* = 0.72, 95% CI [0.55, 0.83], *p* <0.001, *n* = 50).

Full correlation matrices for all study variables within each sample are provided in the Supplement (Table S1–S3).

### Convergent and discriminant validity: comparison of GEEE and CEQ

The expectation of improvement measured by the GEEE was strongly correlated with the CEQ credibility scale (in psychotherapy patients *r* = 0.57, 95% CI [0.42, 0.69], *p* <0.001, *n* = 102, patients on a psychotherapy waitlist *r* = 0.40, 95% CI [0.20, 0.57], *p* <0.001, *n* = 83, and former patients with self-reported mental health issues *r* = 0.56, 95% CI [0.46, 0.64], *p* <0.001, *n* = 219). A similar pattern was observed for the CEQ expectancy scale, with correlations of *r* = 0.63 (95% CI [0.50, 0.74], *p* <0.001, *n* = 102) in psychotherapy patients, *r* = 0.52 (95% CI [0.35, 0.66], *p* <0.001, *n* = 83) in waitlist patients, and *r* = 0.69 (95% CI [0.61, 0.75], *p* <0.001, *n* = 219) in former patients. These results suggest a robust convergence between the GEEE improvement dimension and established CEQ measures of positive treatment expectations. By contrast, expectations of worsening did not show significant associations with CEQ scales in psychotherapy or waitlist patients. In the former patient sample, however, expectation of worsening was negatively correlated with CEQ credibility (*r* = – 0.23, 95% CI [–0.35, − 0.10], *p* <0.001, *n* = 219) and CEQ expectancy (*r* = – 0.19, 95% CI [–0.31, − 0.06], *p* = 0.004, *n* = 219). Expectations of side effects were not significantly correlated with either CEQ scale in any sample, confirming that the CEQ-items do not cover this aspect.

### Associations between depressive symptoms and expectations

Within the psychotherapy sample, participants reported a mean PHQ-9 score of 11.0 (*SD* = 5.60), which falls within the moderate depression range. Analyses showed that depressive symptom severity was negatively correlated with expectations of improvement (*r* = – 0.25, 95% CI [–0.43, − 0.05], *p* = 0.014, *n* = 102), indicating that higher levels of depression were associated with lower expectations of benefit from psychotherapy. By contrast, depressive symptoms were positively correlated with expectations of worsening (*r* = 0.20, 95% CI [0.00, 0.38], *p* = 0.048, *n* = 102) and with expectations of side effects (*r* = 0.20, 95% CI [0.00, 0.38], *p* = 0.048, *n* = 102).

Depressive symptoms were also associated with prior treatment experiences: PHQ-9 scores correlated positively with prior experiences of worsening (*r* = 0.40, 95% CI [0.21, 0.56], *p* <0.001, *n* = 60) and with prior side effect experiences (*r* = 0.32, 95% CI [0.07, 0.53], *p* = 0.014, *n* = 60). Regarding current treatment effects, depressive symptoms were negatively correlated with current improvement (*r* = – 0.34, 95% CI [–0.50, − 0.16], *p* <0.001, *n* = 102) and positively correlated with current worsening (*r* = 0.24, 95% CI [0.05, 0.42], *p* = 0.012, *n* = 102).

## Discussion

The present study provides evidence that expectations of improvement, worsening, and side effects, as assessed by the GEEE, represent related but distinct dimensions across psychotherapy patients, patients awaiting psychotherapy, and former patients with current mental health problems.

Between-group comparisons revealed only minor differences across the three samples. Expectations of improvement and worsening were similar, whereas former psychotherapy patients reported slightly higher side-effect expectations and lower treatment credibility. These differences may reflect self-selection processes, whereby individuals with greater side-effect concerns are less likely to reenter treatment, or the possibility that patients currently engaged in psychotherapy expect fewer potential side effects. This pattern of partial differentiation supports the validity of the GEEE: group differences emerged where theoretically expected, while similar levels of improvement and worsening expectations across samples likely reflect that these core expectations are shaped by broader factors such as cultural attitudes toward psychotherapy, not only by direct treatment exposure.

Our findings support the view that the distinction between positive and negative expectations is empirically supported in our samples. Improvement expectations were largely independent of worsening and side effect expectations, underscoring that positive expectations about therapy outcome are not simply the inverse of expecting a worsening of outcomes. At the same time, worsening and side effect expectations showed substantial overlap, suggesting a shared perception of vulnerability to adverse outcomes in psychotherapy. Across samples, expectations showed consistent associations with corresponding prior and, where applicable, current treatment experiences, underscoring that expectations are substantially intertwined with and shaped by personal treatment trajectories and recent therapeutic experiences.

Associations with depressive symptoms further highlight the potential importance of differentiating treatment expectations. Higher levels of depression were associated with lower improvement expectations and with higher worsening and side effect expectations. However, the direction of these associations is likely multifactorial. Negative treatment experiences may contribute to both higher symptom severity and more pessimistic expectations; conversely, depression itself may bias recall and anticipation toward negative outcomes. It is also possible that unfavorable expectations exacerbate depressive symptoms by reducing engagement with or benefit from treatment. Thus, the observed correlations should not be interpreted as evidence of a single causal pathway but rather as reflecting a complex interplay between depression severity, prior experiences, and expectation formation. Comparisons of correlations across the three samples showed that the dimensional structure of the GEEE was largely stable, with only minor variation. To quantify this similarity, we defined comparable correlation patterns a priori as differences between corresponding coefficients across groups not exceeding Δr = 0.20^46^. According to this criterion, the correlational structure appears similar across samples, though this threshold remains an approximate benchmark. However, in the former patient group only, a small positive correlation between expectations of improvement and expectations of worsening emerged, possibly reflecting ambivalence about treatment outcomes. This ambivalence may partly arise from the link between improvement expectations and prior experiences of worsening during earlier treatment that also only occurred in the former patient sample. Given that this group is no longer actively engaged in psychotherapy, the observed small correlation should be interpreted with caution and in the context of their past-treatment status.

Convergent and discriminant validity analyses further underscored the added value of the GEEE. Improvement expectations correlated strongly with CEQ credibility and expectancy, confirming overlap with established measures of positive expectations. By contrast, worsening and side effect expectations showed no or negative correlations with CEQ subscales, supporting the view that they represent distinct aspects of treatment expectations not captured by existing measures.

Previous research on treatment expectations in psychotherapy has primarily relied on the CEQ, which has become the standard measure in this field. However, the CEQ focuses almost exclusively on positive expectations and does not differentiate between expectations of improvement, worsening, and side effects. The more recently developed TEX-Q provides a multidimensional framework that includes both positive and negative expectations, yet its application has so far been concentrated on medical treatments such as oncology and rehabilitation, with little evidence from psychotherapy contexts^17,18^. The GEEE complements these approaches by offering a concise and generic assessment that captures not only expectations but also prior treatment experiences, a factor that is conceptually central for the formation and modification of expectations. This makes the GEEE particularly suitable for studying long-term and longitudinal therapeutic processes, where expectations evolve dynamically in response to treatment experiences. Experimental studies further demonstrate the clinical relevance of measuring both positive and negative expectations: for example, therapist warmth and competence have been shown to increase positive outcome expectations and strengthen the alliance^47^. Our results extend this work by showing that expectations of worsening and side effects can be assessed alongside positive expectations and that they represent empirically distinct dimensions. The principal added value of the GEEE lies in its measurement architecture, namely a parallel and economical structure for jointly assessing prior experiences, expectations, and effects across the same domains over time, rather than in proposing an entirely new construct beyond established expectancy measures. Its construct-level contribution is more bound but still meaningful: by distinguishing expectations of worsening from expectations of side effects, two facets not assessed by the CEQ and not differentiated in this way by the TEX-Q, the GEEE provides a finer resolution of negative expectations than existing brief measures. The empirical separability of these facets across three samples supports treating them as distinct dimensions rather than as a single negative-expectation factor. Although perhaps surprising at first glance, the independence of positive and negative expectations underscores the inherent complexity and ambiguity of human cognition, whereby individuals can simultaneously maintain hope and dwell on possible negative outcomes.

The interpretation of side effects in the context of psychotherapy warrants brief consideration. Unlike pharmacological side effects, side effects of psychotherapy are not easily delineated and may encompass a broad range of biopsychosocial costs^48^. Empirical work has documented that side effects of psychotherapy include not only symptom-related phenomena such as distress, fatigue, or temporary increases in rumination, but also impairments of social life, including strains in family relationships and interpersonal tensions arising from changes in perspective and behavior^49^. At the same time, side effects is not simply a pharmacological analogy but a defined construct in the psychotherapy adverse-event literature, where it refers to unfavorable changes arising from correctly applied treatment, as distinct from adverse events due to malpractice or unethical conduct^21,50^. This category can be empirically differentiated from the others^51^ and is operationalized in dedicated instruments such as the Side Effects of Psychotherapy Scale^52^ and the side-effects subscale of the Inventory for the balanced assessment of Negative Effects of Psychotherapy (INEP)^53^. We acknowledge that the field also uses the broader heading of negative effects^22^, and that the pharmacological analogy has evident limits in the psychotherapeutic context. We retain the term side effects because it is the validated wording of the GEEE item and preserves comparability with the instrument’s prior applications. The present results suggest that, despite this conceptually wide range, participants responded to the side-effect item in a coherent and discriminating manner. The correlation patterns involving side-effect expectations replicated across three independent samples differing in treatment phase, and side-effect expectations showed consistently low associations with expectations of worsening, indicating that participants clearly distinguished therapy-related adverse experiences from symptom deterioration. Capturing this dimension is clinically meaningful given established links between side-effect expectations and nocebo effects, treatment adherence, and dropout.

### Strengths and limitations

A central strength of this study lies in its novelty and scope. To our knowledge, it is the first to examine the three GEEE dimensions of improvement, worsening, and side effects with regard to psychotherapy across multiple samples. By including three independent samples of psychotherapy patients, prescreened patients awaiting psychotherapy, and former patients with self-reported mental health issues, we were able to show that these dimensions behave in a similar way across different populations. Another strength is the direct comparison with the CEQ, which demonstrated that the GEEE improvement dimension overlaps with CEQ scores, while expectations of worsening and side effects represent distinct facets not captured by the CEQ. Moreover, the results provide evidence that the three expectation domains can be meaningfully distinguished, supporting the construct validity of the GEEE. Finally, the study addresses an important gap in the field by focusing on negative expectations such as worsening and side effects, which are rarely assessed but highly relevant in clinical practice.

At the same time, some limitations should be noted. The cross-sectional design precludes conclusions about predictive validity; longitudinal research is needed to examine whether the three expectation dimensions predict treatment processes or outcomes and how treatment expectations change over time. While the inclusion of three distinct samples strengthens the study, it should be acknowledged that the former patient sample relied on self-reported mental health problems rather than independently verified diagnoses. Additionally, analyses of associations between expectations and symptom severity were restricted to depressive symptoms in the psychotherapy sample; generalization to other diagnostic groups requires further empirical investigation. Furthermore, the GEEE currently lacks established norm values, cut-off scores, or interpretive benchmarks, which limits the clinical interpretability of individual scores; and while gender and age were examined as covariates and did not affect the pattern of group differences (see Supplement), the role of education on GEEE scores remains to be systematically examined.

The comparability of correlation patterns across samples was defined pragmatically using Cohen’s^46^ effect size framework. This operational definition provides a transparent criterion suited to the exploratory nature of this work, although it remains an approximate rather than a formal test of invariance. Furthermore, the GEEE assesses side-effect expectations in a global single-item format, which leaves the specific content of these expectations open to individual interpretation. This was a deliberate choice consistent with the descriptive aim of the present study, but future research using item-level probing or qualitative methods could clarify the specific phenomena patients have in mind when rating this dimension.

### Clinical implications and future directions

The present findings open up numerous avenues for future research in psychotherapy and related fields. Key open questions concern the temporal dynamics of treatment expectations. How do expectations of improvement, worsening, and side effects change over the course of therapy? How do they interact with prior and subsequent health outcomes, and which aspects of the therapeutic process such as the quality of the alliance, perceived warmth, and therapist competence influence these relationships? Addressing these questions will require longitudinal and experimental studies that link expectation dynamics to behavioral and clinical outcomes. Moreover, future research should evaluate the applicability of the GEEE across different diagnostic groups and treatment contexts, and test whether its brevity and multidimensional structure make it suitable as a personalized screening or monitoring tool in routine clinical practice.

From a clinical perspective, patients’ early treatment expectations are established predictors of psychotherapy outcomes. Meta-analyses have shown that more positive expectations are reliably associated with better results^4,5^. Conversely, low or negative expectations can increase the risk of disengagement or unfavorable outcomes, emphasizing the importance of systematic assessment and early therapeutic addressing of maladaptive expectations. Expectation-focused psychotherapeutic approaches (EFPI) provide promising strategies to strengthen adaptive expectations through systematic expectation violations and targeted cognitive interventions^54,55^. By identifying individual expectation profiles, clinicians can tailor communication and intervention strategies to foster positive expectations while reducing negative ones, ultimately enhancing engagement and treatment success.

## Conclusion

This study provides initial evidence that the GEEE captures three distinct dimensions of treatment expectations (improvement, worsening, and side effects) across psychotherapy patients, patients awaiting treatment, and former patients with self-reported mental health problems. With less items, the improvement dimension aligns closely with the CEQ, while expectations of worsening and side effects emerge as unique facets that are often overlooked. Associations with depressive symptoms further underscore the potential importance of differentiating these dimensions. By offering a brief, parallel measurement architecture that jointly assesses related expectation domains, and by differentiating worsening from side-effect expectations more finely than existing brief measures, the GEEE fills an important gap and opens new avenues for both research and practice.

## Supplementary Information

Below is the link to the electronic supplementary material.

Supplementary Material 1

Supplementary Material 2

## Data Availability

All data and codes used for the analyses described will be made available upon reasonable request from the corresponding author.
