# Supplementary material for: Distinct positive and negative dimensions of psychotherapy treatment expectations across three independent samples
Source: Sci Rep. 2026 Jul 3;16:20551. doi: 10.1038/s41598-026-60951-7 (PMC13332196; doi:10.1038/s41598-026-60951-7)
Supplement: Supplementary file 1 — Supplementary Material 1 [file 41598_2026_60951_MOESM1_ESM.docx]

| **Table S1. Correlation Matrix (psychotherapy patients)** | | | | | | | | | | | | |
| --- | --- | --- | --- | --- | --- | --- | --- | --- | --- | --- | --- | --- |
| Variable | Expectation of improvement | Expectation of worsening | Expectation of side effects | CEQ credibility | CEQ expectancy | Prior improvement experiences | Prior worsening experiences | Prior side-effect experiences | PHQ-9 depressive symptoms | Current improvement effects | Current worsening effects | Current side-effect effects |
| Expectation of improvement | 1.00 |  |  |  |  |  |  |  |  |  |  |  |
| Expectation of worsening | -0.02 | 1.00 |  |  |  |  |  |  |  |  |  |  |
| Expectation of side effects | -0.19 | 0.49*** | 1.00 |  |  |  |  |  |  |  |  |  |
| CEQ credibility | 0.57*** | -0.09 | 0.00 | 1.00 |  |  |  |  |  |  |  |  |
| CEQ expectancy | 0.63*** | -0.12 | 0.00 | 0.70*** | 1.00 |  |  |  |  |  |  |  |
| Prior improvement experiences | 0.26* | 0.14 | -0.02 | 0.13 | 0.16 | 1.00 |  |  |  |  |  |  |
| Prior worsening experiences | 0.10 | 0.37** | 0.25 | 0.00 | 0.01 | -0.42*** | 1.00 |  |  |  |  |  |
| Prior side-effect experiences | -0.03 | 0.14 | 0.29* | -0.02 | -0.03 | -0.32* | 0.74*** | 1.00 |  |  |  |  |
| PHQ-9 depressive symptoms | -0.25* | 0.20* | 0.20* | -0.33*** | -0.42*** | -0.19 | 0.40** | 0.32* | 1.00 |  |  |  |
| Current improvement effects | 0.22* | 0.14 | -0.03 | 0.26** | 0.37*** | -0.08 | 0.23 | 0.25 | -0.34*** | 1.00 |  |  |
| Current worsening effects | 0.00 | 0.47*** | 0.25** | -0.13 | -0.07 | 0.04 | 0.34** | 0.34** | 0.24* | -0.10 | 1.00 |  |
| Current side-effect effects | -0.07 | 0.47*** | 0.46*** | -0.08 | -0.03 | 0.11 | 0.12 | 0.37** | 0.09 | 0.21* | 0.56*** | 1.00 |

| **Pairwise Ns** | | | | | | | | | | | | |
| --- | --- | --- | --- | --- | --- | --- | --- | --- | --- | --- | --- | --- |
| Variable | Expectation of improvement | Expectation of worsening | Expectation of side effects | CEQ credibility | CEQ expectancy | Prior improvement experiences | Prior worsening experiences | Prior side-effect experiences | PHQ-9 depressive symptoms | Current improvement effects | Current worsening effects | Current side-effect effects |
| Expectation of improvement | - |  |  |  |  |  |  |  |  |  |  |  |
| Expectation of worsening | 102 | - |  |  |  |  |  |  |  |  |  |  |
| Expectation of side effects | 102 | 102 | - |  |  |  |  |  |  |  |  |  |
| CEQ credibility | 102 | 102 | 102 | - |  |  |  |  |  |  |  |  |
| CEQ expectancy | 102 | 102 | 102 | 102 | - |  |  |  |  |  |  |  |
| Prior improvement experiences | 60 | 60 | 60 | 60 | 60 | - |  |  |  |  |  |  |
| Prior worsening experiences | 60 | 60 | 60 | 60 | 60 | 60 | - |  |  |  |  |  |
| Prior side-effect experiences | 60 | 60 | 60 | 60 | 60 | 60 | 60 | - |  |  |  |  |
| PHQ-9 depressive symptoms | 102 | 102 | 102 | 102 | 102 | 60 | 60 | 60 | - |  |  |  |
| Current improvement effects | 102 | 102 | 102 | 102 | 102 | 60 | 60 | 60 | 102 | - |  |  |
| Current worsening effects | 102 | 102 | 102 | 102 | 102 | 60 | 60 | 60 | 102 | 102 | - |  |
| Current side-effect effects | 102 | 102 | 102 | 102 | 102 | 60 | 60 | 60 | 102 | 102 | 102 | - |

**Note.** Values are Pearson correlations (*r*) shown in the lower triangle; diagonal entries = 1.00; upper triangle omitted for readability. Significance is coded as *p* < .05 = *, *p* < .01 = **, *p* < .001 = ***. Pairwise sample sizes (*n*) for each correlation are reported in the accompanying “Pairwise Ns” table. Variables: Expectation of improvement, Expectation of worsening, Expectation of side effects; Prior improvement experiences, Prior worsening experiences, Prior side-effect experiences; Current improvement effects, Current worsening effects, Current side-effect effects (patients only); CEQ credibility, CEQ expectancy; PHQ-9 depressive symptoms (if available).

| **Table S2. Correlation Matrix (patients on a psychotherapy waitlist)** | | | | | | | | |
| --- | --- | --- | --- | --- | --- | --- | --- | --- |
| Variable | Expectation of improvement | Expectation of worsening | Expectation of side effects | CEQ credibility | CEQ expectancy | Prior improvement experiences | Prior worsening experiences | Prior side-effect experiences |
| Expectation of improvement | 1.00 |  |  |  |  |  |  |  |
| Expectation of worsening | 0.08 | 1.00 |  |  |  |  |  |  |
| Expectation of side effects | 0.02 | 0.55*** | 1.00 |  |  |  |  |  |
| CEQ credibility | 0.40*** | 0.15 | 0.11 | 1.00 |  |  |  |  |
| CEQ expectancy | 0.52*** | 0.16 | 0.08 | 0.59*** | 1.00 |  |  |  |
| Prior improvement experiences | 0.36** | 0.13 | 0.11 | 0.34* | 0.27 | 1.00 |  |  |
| Prior worsening experiences | 0.05 | 0.59*** | 0.51*** | -0.11 | 0.17 | 0.06 | 1.00 |  |
| Prior side-effect experiences | 0.20 | 0.30* | 0.42** | 0.25 | 0.25 | 0.15 | 0.72*** | 1.00 |

| **Pairwise Ns** | | | | | | | | |
| --- | --- | --- | --- | --- | --- | --- | --- | --- |
| Variable | Expectation of improvement | Expectation of worsening | Expectation of side effects | CEQ credibility | CEQ expectancy | Prior improvement experiences | Prior worsening experiences | Prior side-effect experiences |
| Expectation of improvement | - |  |  |  |  |  |  |  |
| Expectation of worsening | 83 | - |  |  |  |  |  |  |
| Expectation of side effects | 83 | 83 | - |  |  |  |  |  |
| CEQ credibility | 83 | 83 | 83 | - |  |  |  |  |
| CEQ expectancy | 83 | 83 | 83 | 83 | - |  |  |  |
| Prior improvement experiences | 50 | 50 | 50 | 50 | 50 | - |  |  |
| Prior worsening experiences | 50 | 50 | 50 | 50 | 50 | 50 | - |  |
| Prior side-effect experiences | 50 | 50 | 50 | 50 | 50 | 50 | 50 | - |

**Note**. Values are Pearson correlations (r) shown in the lower triangle; diagonal entries = 1.00; upper triangle omitted for readability. Significance is coded as p < .05 = *, p < .01 = **, p < .001 = ***. Pairwise sample sizes (n) for each correlation are reported in the accompanying “Pairwise Ns” table. Variables: Expectation of improvement, Expectation of worsening, Expectation of side effects; Prior improvement experiences, Prior worsening experiences, Prior side-effect experiences; Current improvement effects, Current worsening effects, Current side-effect effects (patients only); CEQ credibility, CEQ expectancy; PHQ-9 depressive symptoms (if available).

| **Table S3. Correlation Matrix (former psychotherapy patients with current problems)** | | | | | | | | |
| --- | --- | --- | --- | --- | --- | --- | --- | --- |
| Variable | Expectation of improvement | Expectation of worsening | Expectation of side effects | CEQ credibility | CEQ expectancy | Prior improvement experiences | Prior worsening experiences | Prior side-effect experiences |
| Expectation of improvement | 1.00 |  |  |  |  |  |  |  |
| Expectation of worsening | -0.20** | 1.00 |  |  |  |  |  |  |
| Expectation of side effects | 0.04 | 0.55*** | 1.00 |  |  |  |  |  |
| CEQ credibility | 0.56*** | -0.23*** | -0.02 | 1.00 |  |  |  |  |
| CEQ expectancy | 0.69*** | -0.19** | 0.03 | 0.67*** | 1.00 |  |  |  |
| Prior improvement experiences | 0.50*** | -0.26*** | -0.12 | 0.44*** | 0.53*** | 1.00 |  |  |
| Prior worsening experiences | -0.15* | 0.60*** | 0.47*** | -0.12 | -0.15* | -0.21** | 1.00 |  |
| Prior side-effect experiences | -0.07 | 0.51*** | 0.70*** | -0.07 | -0.03 | -0.05 | 0.63*** | 1.00 |

| **Pairwise Ns** | | | | | | | | |
| --- | --- | --- | --- | --- | --- | --- | --- | --- |
| Variable | Expectation of improvement | Expectation of worsening | Expectation of side effects | CEQ credibility | CEQ expectancy | Prior improvement experiences | Prior worsening experiences | Prior side-effect experiences |
| Expectation of improvement | - |  |  |  |  |  |  |  |
| Expectation of worsening | 219 | - |  |  |  |  |  |  |
| Expectation of side effects | 219 | 219 | - |  |  |  |  |  |
| CEQ credibility | 219 | 219 | 219 | - |  |  |  |  |
| CEQ expectancy | 219 | 219 | 219 | 219 | - |  |  |  |
| Prior improvement experiences | 219 | 219 | 219 | 219 | 219 | - |  |  |
| Prior worsening experiences | 219 | 219 | 219 | 219 | 219 | 219 | - |  |
| Prior side-effect experiences | 219 | 219 | 219 | 219 | 219 | 219 | 219 | - |

**Note.** Values are Pearson correlations (*r*) shown in the lower triangle; diagonal entries = 1.00; upper triangle omitted for readability. Significance is coded as *p* < .05 = *, *p* < .01 = **, *p* < .001 = ***. Pairwise sample sizes (*n*) for each correlation are reported in the accompanying “Pairwise Ns” table. Variables: Expectation of improvement, Expectation of worsening, Expectation of side effects; Prior improvement experiences, Prior worsening experiences, Prior side-effect experiences; Current improvement effects, Current worsening effects, Current side-effect effects (patients only); CEQ credibility, CEQ expectancy; PHQ-9 depressive symptoms (if available).
