# Supplementary material for: Distinct positive and negative dimensions of psychotherapy treatment expectations across three independent samples
Source: Sci Rep. 2026 Jul 3;16:20551. doi: 10.1038/s41598-026-60951-7 (PMC13332196; doi:10.1038/s41598-026-60951-7)
Supplement: Supplementary file 2 — Supplementary Material 2 [file 41598_2026_60951_MOESM2_ESM.docx]

**Supplement: ANCOVA Results for Between-Group Comparisons**

*Covariate adjustment for gender and age*

**Table S1. Between-Group Comparisons: Unadjusted and Covariate-Adjusted Group Means**

| **Outcome** | **Group** | **n** | **M (raw)** | **M (adjusted)** | **SD** | **p (ANOVA)** | **p (ANCOVA)** |
| --- | --- | --- | --- | --- | --- | --- | --- |
| **Expectation of improvement** | Psychotherapy patients | 101 | 8.41 | 8.42 | 1.67 | .332 | .296 |
|  | Waitlist patients | 83 | 8.11 | 8.15 | 1.61 |  |  |
|  | Former patients | 219 | 8.10 | 8.09 | 1.85 |  |  |
| **Expectation of worsening** | Psychotherapy patients | 101 | 2.06 | 2.37 | 1.57 | .187 | .170 |
|  | Waitlist patients | 83 | 2.57 | 2.88 | 2.17 |  |  |
|  | Former patients | 219 | 2.32 | 2.65 | 1.82 |  |  |
| **Expectation of side effects** | Psychotherapy patients | 101 | 3.47 | 3.69 | 2.31 | .016 | .022 |
|  | Waitlist patients | 83 | 3.69 | 3.92 | 2.63 |  |  |
|  | Former patients | 219 | 4.27 | 4.48 | 2.52 |  |  |
| **CEQ credibility** | Psychotherapy patients | 101 | 0.57 | 0.32 | 1.90 | < .001 | < .001 |
|  | Waitlist patients | 83 | 0.56 | 0.35 | 1.40 |  |  |
|  | Former patients | 219 | -0.47 | -0.81 | 3.02 |  |  |
| **CEQ expectancy** | Psychotherapy patients | 101 | 0.01 | 0.13 | 2.25 | .795 | .939 |
|  | Waitlist patients | 83 | -0.16 | 0.03 | 2.35 |  |  |
|  | Former patients | 219 | 0.07 | 0.15 | 2.87 |  |  |

*Note.* M (raw) = unadjusted group mean on the 0–10 GEEE rating scale. M (adjusted) = estimated marginal mean from ANCOVA with gender and age as covariates. SD = standard deviation of raw scores. p (ANOVA) = omnibus p-value from one-way ANOVA without covariates (as reported in main text). p (ANCOVA) = omnibus p-value from Type III ANCOVA. CEQ = Credibility/Expectancy Questionnaire; CEQ scores are z-standardized. Sample sizes reflect the ANCOVA analytic sample after listwise deletion of one psychotherapy participant with missing covariate data (analytic N = 403).

**Table S2. Tukey-Adjusted Post-Hoc Comparisons for Significant Outcomes (ANCOVA)**

*Only outcomes with omnibus p < .05 in both ANOVA and ANCOVA are shown (Expectation of side effects; CEQ credibility).*

| **Outcome** | **Contrast** | **Difference** | **95% CI** | **t** | **p** |
| --- | --- | --- | --- | --- | --- |
| **Expectation of side effects** | Psychotherapy patients vs. Waitlist patients | -0.23 | [-1.11, 0.64] | -0.62 | .807 |
|  | Psychotherapy patients vs. Former patients | -0.79 | [-1.50, -0.08] | -2.62 | **.025** |
|  | Waitlist patients vs. Former patients | -0.56 | [-1.34, 0.22] | -1.70 | .208 |
| **CEQ credibility** | Psychotherapy patients vs. Waitlist patients | -0.03 | [-0.90, 0.84] | -0.08 | .996 |
|  | Psychotherapy patients vs. Former patients | 1.13 | [0.43, 1.84] | 3.77 | **< .001** |
|  | Waitlist patients vs. Former patients | 1.17 | [0.39, 1.94] | 3.54 | **.001** |

*Note.* Difference = estimated marginal mean difference (first group minus second group) adjusted for gender and age. 95% CI = Tukey-adjusted confidence interval. Bold p-values indicate significance at p < .05.
